# The unique contributions of depression and anxiety to suicidal ideation among Nigerian secondary school students: a cross-sectional survey

**DOI:** 10.4314/ahs.v23i4.57

**Published:** 2023-12

**Authors:** Awoere T Chinawa, Edmund N Ossai, Josephat M Chinawa, Paul C Odinka, Obinna C Nduagubam, Jaclyn I Odinka, Ann E Aronu

**Affiliations:** 1 Consultant Community Physician and Senior Lecturer Enugu State University Teaching hospital, Enugu State, Nigeria; 2 Department of Community Medicine, College of Health Sciences, Ebonyi State University Abakaliki, Nigeria; 3 Department of Paediatrics, College of Medicine, University of Nigeria Enugu Campus, Nigeria; 4 Department of Psychological Medicine, University of Nigeria Enugu Campus, Nigeria; 5 Department of Paediatrics, Enugu State University Teaching hospital, Enugu State, Nigeria; 6 Social Sciences Unit, School of General Studies, University of Nigeria Nsukka, Nigeria

**Keywords:** Suicidal ideation, depression, anxiety, adolescents, secondary school, Enugu State, Nigeria

## Abstract

**Background:**

It is important to establish the pattern of suicidal ideation among adolescents with depression and anxiety. This will help the schools prevent possible complete suicide among these groups of adolescents' trough counseling and education.

**Objectives:**

This work is therefore aimed at determining the effects of depression and anxiety on adolescent suicide ideation and factors that predict suicidal ideation among adolescents attending secondary school in southeast Nigeria.

**Methods:**

A cross-sectional study among 450 secondary school adolescents in Enugu state, Nigeria. Information was obtained using the Columbia suicide severity rating scale and the Hospital Anxiety and Depression Scale (HADS). Chi-square test, correlation analysis and Hierarchical logistic regression were used in the analysis, and the level of statistical significance was determined using a P-value of <0.05.

**Results:**

The prevalence of anxiety was 36.4% while that of depression was 30.7%. The prevalence of suicidal ideation was 8.4%. The prevalence of suicidal ideation among adolescents who were <15 years was 8.0% while those who were between 15 – 18 years was 13.5% and those more than 19 years of age was 22.5%. The prevalence of suicidal ideation among adolescents with depression is 21.7% and 78.3 % for those without depression while the prevalence of suicidal ideation among adolescents with anxiety is 20.1% and 79.9 % for those without anxiety. The prevalence of suicidal ideation among the poorest half was 16.4% and 10.2% in the richer half. There was a higher prevalence of suicidal ideation among the urban dwellers (53.3%) than the rural dwellers (46.7%).

There was a positive correlation between suicide and age in years and this was found to be statistically significant, (n=450, r=0.107, p=0.023). A significantly higher proportion of respondents who had depression, 21.7% had suicidal ideation when compared with those who were not, (*χ*^2^=12.170, p<0.001). Similarly, a significantly higher proportion of respondents who had an anxiety disorder, (20.1%) had suicidal ideation when compared with those who were not, 9.4%, (*χ*^2^=10.291, p=0.001). The respondents who were 19 years and above had increased suicidal ideation, and the difference in proportions was found to be statistically significant, (*χ*^2^=6.440, p=0.040). A significantly higher proportion of females, 16.6% had suicidal intentions when compared with the males, 7.1%, (*χ*^2^=7.958, p=0.005).

**Conclusion:**

There is an increasing prevalence of suicidal ideation among adolescents with a preponderance of older age and female gender. There is a link between depression/anxiety and suicidal ideation. The prevalence of suicidal ideation among the poorest half was higher than that of the richer half. There was a higher prevalence of suicidal ideation among the urban dwellers than the rural dwellers.

## Introduction

Adolescence is a crucial period in which individuals begin their transition from childhood to adulthood.^1^ Mental health conditions have raised serious concerns for adolescents. For instance, it is estimated that 1 in 7 (14%) of adolescents aged 10-19 years have mental health conditions, however, these were largely unrecognized and untreated^2^

A retrospective survey in the USA in 2003 showed that almost fifty percent of all mental health conditions start by 14 years of age, regrettably the majority of them were either undetected or untreated.^3^ Though there is a rising awareness of mental health of adults, this is not so in the adolescent child. Depression and suicidal thoughts among other mental problems are the major cause of morbidity according to the Nationwide survey data on more than 230,000 USA adolescents over 13 years.^4^ The relevance of brain changes in early-onset individuals with depression, particularly among young adolescents cannot be overemphasized.^5^ More abnormalities have been documented in the brains of adolescents with bipolar than unipolar depression.^5^ For instance, a systematic review of several works done between 1980 and 2013 reported white and gray matter changes in the adolescent with depression. 5 Notable pathological changes were seen in the brains of adolescents with bipolar than unipolar depression. 5 Reductions in the volume of basal ganglia and the hippocampus were seen especially among the adolescents with unipolar depression, while reduced corpus callosum volume were seen in adolescents with bipolar depression.^5^

Furthermore, there is a link between adolescent depression and negative clinical outcomes, especially particularly suicide ideation which is frequently under-reported. 6 It is interesting to note that more than 2% of the traffic accidents are suicide behaviors.^6^ This phenomenon may be under-reported considering that suicides by car accidents may be reported as accidental in the national statistics. 6 This was buttressed in the reportage of Pompili 6 et al. Nevertheless, the link between accident-proneness and suicidal ideation and other self-destructive impulses remains conjectural.^6^

Similarly, anxiety disorders and suicide ideation (attempts, plans of completed suicide) are both major topical health issues in adolescents.^7^,^8^ There remains an independent association between anxiety and suicidality.^8^ Beyond the existing link between demographic factors and depression, it is not well documented in literature if anxiety disorders could cause an increased risk for suicidality in adolescents.^9^ Suicidal ideation is of major public health importance. 10 It may present as passive ideation (wants to be dead) to active ideation (wanting to kill oneself).^11^-^13^

Suicidal ideation in the adolescent occurs with increasing age.^12^ Worldwide prevalence rates were 0.5 for females and 0.9 for males in the age of 5-14-year-olds, and 12.0 for females and 14.2 for males in the age 15-24-year-old per 100000, respectively.^13^ There is a male preponderance except in China, Cuba, Ecuador, El Salvador , and Sri Lanka, where the female suicide rate was higher than the male.^14^ Data from African countries revealed the prevalence of adolescent suicide as 3.3 per 100,000.^15^ This is in tandem with the estimate of 49,558 suicides seen in a recent study 2010.^16^ It is noted that about fifty percent of adolescents who have committed suicide had received psychological attention recently.^16^ Notable risk factors for suicide include depression, anxiety, substance abuse and a history of suicide attempts. In southeast Nigeria, the prevalence of anxiety and depression is quite high, however, some of these adolescents may have thoughts of suicide without being noticed. If this is not attended to, complete suicide becomes the norm. It is important to establish the pattern and burden of suicidal ideation among these groups of adolescents. Screening for suicide ideation among these groups of adolescents will help to avert suicide in the nearest future. Though systematic reviews conducted in the united state have shown insufficient evidence to support the screening for suicidal ideation among adolescents without mental disorders or a history of attempted suicide, yet evidence showed that screening for suicidal ideation had assisted in identifying adolescents who are prone to suicide.^17^,^18^

This study is crucial in that it creates a template for the development of intervention-type actions which will help to identify and curb suicidal ideation among the adolescents. Several retrospective studies in Nigeria have documented the prevalence of complete suicide in adolescents but much has not been done to determine adolescents with suicidal ideation or who had a tendency for suicidal ideation. Besides, most studies on this topic were clustered among the adult population.

This present study is the first one on this topic done at the University of Nigeria Ituku- Ozalla. This work is therefore aimed at determining the effects of depression and anxiety on adolescent suicide ideation and factors that predict suicidal ideation among adolescents attending secondary school in southeast Nigeria.

## Methods

### Study area and population

This is a cross-sectional study that enrolled a total of four hundred and fifty secondary school adolescents undertaken in six secondary schools from September 2021 to January 2022. A study frame of the six schools selected in the study consists of 3 rural and 3 urban secondary schools.

### Sampling technique

A three-stage sampling technique was used to select the respondents for inclusion in the study. In the first stage, a simple random sampling technique of balloting was used to select two local government areas in the urban and same in the rural areas of the state. There are 17 local government areas in Enugu state of which 5 of the local government areas are designated as urban local government areas. In the second stage, a list of all the secondary schools in the three selected local government areas was made and using a simple random sampling technique of balloting two secondary schools were selected in each of the local government areas. Each school contributed at least 55 respondents to the study. The schools were stratified into 3 mixed schools, and 3 same-sex schools. In each of the selected schools, the number of students in the senior secondary classes of senior secondary 1 and 2 classes were made and this served as the sampling frame. Dividing this number by 55, a sampling interval was obtained. The first respondent was selected using a simple random sampling technique of balloting after which the interval was applied.

### Study design

The present study is a cross-sectional study among secondary school adolescents who attended secondary schools in both rural and urban towns in Enugu city.

### Inclusion criteria

Secondary school adolescents who had no known psychiatric illness or who have not been diagnosed with any psychiatric illness before the beginning of the study.

### Exclusion criteria

Secondary school adolescents with any known or diagnosed psychiatric illness.

### Study instrument

The suicidal ideation was assessed using the Columbian suicide severity rating scale.^19^ This is a validated questionnaire made up of six variables. Each of the six variables had a yes and no response option. A yes response attracted a score of one while no response was scored zero. The total score for each of the respondents was determined and categorized as follows; a total score of zero denoted no suicide risk. A score of 1-2 meant low risk, a score of 3-4 moderate risk while 4-6 denoted severe suicidal ideation. The suicidal ideation was further dichotomized into No suicidal ideation and suicidal ideation. The Columbia suicide severity rating scale used for this study was written in English Language and this was explained in detail to the adolescents. The Questionnaires were administered by trained research assistants.

The Hospital Anxiety and Depression Scale (HADS) questionnaire were used to assess anxiety and depression.^20^ This is a validated questionnaire made up of fourteen variables, each being a four-point Likert scale with scores of zero to three. Seven of the variables were used to assess anxiety and the other seven were used to determine depression. The total score for the seven variables used to assess anxiety was calculated for each respondent. A total score of 0-7 meant the respondent had no anxiety. A total score of 8-10 meant mild anxiety, 11-14; moderate anxiety and scores of between 15 and 21 denoted severe anxiety. The anxiety variable was further categorized into two, no anxiety and anxiety present. The same method of calculation was used to determine and classify the respondents' responses to the seven variables used to assess depression.

### Sample size estimation

For a 95% confidence level and 5% precision for a population >100,000 to be attained, an estimated 400 secondary school adolescents were used as the minimum sample size. This was derived from the tables of sample sizes by Glenn 21 et al. These tables of sample sizes allow a given combination of precision, confidence level, and variability for different population size to be ascertained. However, a 12% attrition rate was considered and this brought the sample size to approximately 450 secondary school adolescents.

### Method of data collection

Data was collected from the respondents in their various schools. For respondents in the Senior Secondary 2 in the urban area, a self-administered questionnaire was used. However, for respondents in Senior secondary 1 in the urban area and all respondents in the rural areas of the state, the questionnaire was interviewer administered. This approach was used so as to ensure the validity of the results.

### Ethical Considerations

Ethical approval was obtained from the Ethics and Research Committee of our institution while permission was obtained from the principals of all the schools that were included in the study.

### Consent

Oral consent was obtained from all the students who were 18 years and above while assent was obtained from the adolescents who were less than 18 years before questionnaire administration. Only five students who were 18 years and above refused to participate in the study. All the students who their parents or guardians granted informed consent participated in the study.

### Social class estimation

The socioeconomic class of the adolescent's family was calculated using Principal component analysis (PCA) using STATA statistical software version 12. Items such as family ownership of household items; radio, television, refrigerator, car, bicycle and availability of electricity were included. Other variables used in the PCA were type of toilet facility and residential building. Students were asked to identify which of these items were acquired by their parents.

Quartiles were used for a distribution cut-off points with each adolescent assigned the wealth index score of his/her family. The quartiles included Q1=poorest, Q2=the very poor, Q3=the poor and Q4, the least poor. The quartiles were then categorized into two groups, the poorest and very poor were classified as the low socio-economic class while the high socio-economic class included the poor and least poor groups.

### Data analysis

Data entry and analysis were done using IBM Statistical Package for Social Sciences (SPSS) statistical package version 25. Categorical variables were summarized using frequencies and proportions while continuous variables were presented using mean and standard deviation. Chi-square test was used to compare the difference in proportions between two categorical variables. Correlation analysis was used to determine the strength of the linear relationship between two continuous variables. Multivariate analysis using hierarchical binary logistic regression was used to determine the predictors of the outcome variable; suicidal ideation. The level of statistical significance was determined by a P- value of <0.05.

The results of the hierarchical logistic regression analysis were presented using an adjusted odds ratio and 95% confidence interval and the level of statistical significance was determined using a P-value of <0.05. The logistic regression model was fitted using Hosmer-Lemeshow goodness-of-fit.

## Results

Table 1 shows the socio-demographic characteristics of the respondents. The mean age of the respondents was 16.2±1.7 years. The highest proportion of the respondents, 66.0% were in the age group 15-18 years. The majority, 65.6% were females. A higher proportion of the respondents, 53.3% reside in the urban areas.

**Table 1:** Socio-demographic characteristics of the respondents

Variable	Frequency (n=450)	Percent (%)
**Age of respondents**		
Mean± SD	16.2±1.7	
		
**Age of respondents in groups**		
<15 years	100	22.2
15 – 18 years	297	66.0
≥19 years	53	11.8
**Gender**		
Male	155	34.4
Female	295	65.6
**Religion**		
Christianity	418	92.9
Islam	27	6.0
Traditional religion	5	1.1
**Location**		
Urban	240	53.3
Rural	210	46.7
**Educational attainment of Mother**		
No formal education	29	6.4
Primary education	74	16.4
Secondary education	198	44.0
Tertiary education	149	33.1
**Family Socio-economic status**		
Low socio-economic class	225	50.0
High socio-economic class	225	50.0

Table 2 shows the respondents' responses to the Columbia suicide severity rating scale. A minor proportion of the respondents, 8.4% had wished themselves dead or slept and did not wake up. Similarly, a minor proportion of the respondents, 8.7% have actually had thoughts of killing oneself. A very minor proportion of the respondents, 0.9% have started to work out or worked out details of how to kill oneself. Majority of the respondents, 86.7% had no suicidal ideation based on the Columbia suicide severity rating scale while a minor proportion, 1.8% had a high risk.

**Table 2:** Respondents' responses to the Columbia suicide severity rating scale

Variable	Frequency (n=450)	Percent (%)
**Have wished oneself dead or sleep and not wake up**		
Yes	38	8.4
**Have actually had thoughts of killing oneself**		
Yes	39	8.7
**Have had thoughts about how one might do this**		
Yes	12	2.7
**Have had these thoughts and the intention of acting them**		
Yes	9	2.0
**Have started to work out or worked out details of how to kill oneself**		
Yes	4	0.9
**Have ever done anything or started to do anything to end one's life**		
Yes	26	5.8
**Columbia suicide severity rating scale**		
No risk	390	86.7
Low risk	39	8.7
Moderate risk	13	2.9
High risk	8	1.8
**Columbia suicide severity rating scale Categorized**		
No risk	390	86.7
Risk	60	13.3

Table 3 shows assessment of depression using the Hospital Anxiety and Depression Scale. The highest proportion of the respondents, 61.8% do not get very frightened at all, 16.7% get frightened sometimes while a minor proportion, 9.6% gets very frightened. The highest proportion of the respondents, 76.4% do not have palpitations at all, 9.6% have palpitations sometimes while 5.1% definitely have palpitations.

**Table 3:** Assessment of depression using the hospital anxiety and depression scale (n=450)

Variable	Not at all N (%)	No of much N (%)	Yes, sometimes N (%)	Yes definitely N (%)
I get very frightened	278 (61.8)	54 (12.0)	75 (16.7)	43 (9.6)
I feel anxious when I get out of the house	286 (63.6)	48 (10.7)	66 (14.7)	50 (11.1)
I get palpitations	344 (76.4)	40 (8.9)	43 (9.6)	23 (5.1)
I feel scared or frightened	283 (62.9)	59 (13.1)	74 (16.4)	34 (7.6)
I still enjoy the things I used to	125 (27.8)	45 (10.0)	76 (16.9)	204 (45.3)
I am more irritable than usual	305 (67.8)	67 (14.9)	51 (11.3)	27 (6.0)
Worrying thoughts constantly go through my mind	238 (52.9)	35 (7.8)	89 (19.8)	88 (19.6)

Table 4: Assessment of anxiety using the Hospital Anxiety and Depression Scale. The highest proportion of the respondents, 65.6% have never felt miserable or sad, 13.6% feel miserable or sad sometimes while a minor proportion, 8.2% always feel miserable and sad. The highest proportion of the respondents, 64.2% have never felt as if they are slowed down, 15.1% feel slowed down sometimes while about one tenth of the respondents, 10.2% definitely feel as if they are slowed down.

**Table 4:** Assessment of anxiety using the hospital anxiety and depression scale (n=450)

Variable	Not at all N (%)	No of much N (%)	Yes, sometimes N (%)	Yes definitely N (%)
I wake early and then sleep badly the rest of the night	218 (48.4)	51 (11.3)	137 (30.4)	44 (9.8)
I feel miserable and sad	295 (65.6)	57 (12.7)	61 (13.6)	37 (8.2)
I have lost interest in things	306 (68.0)	55 (12.2)	59 (131)	30 (6.7)
I have a good appetite	121 (26.9)	56 (12.4)	74 (16.4)	199 (44.2)
I feel life is not worth living	333 (74.0)	36 (8.0)	49 (10.9)	32 (7.1)
I am restless and can't keep still	316 (70.2)	53 (11.8)	54 (12.0)	27 (6.0)
I feel as if I have slowed down	289 (64.2)	47 (10.4)	68 (15.1)	46 (10.2)

Table 5 shows the prevalence of depression and anxiety among the respondents. More than one- third of the respondents, 36.4% were anxious. Among the respondents who were anxious, the highest proportion, 16.9% were moderately anxious while the least proportion, 4.7% were severely anxious. Less than one-third of the respondents, 30.7% were depressed. Among the respondents who were depressed, the highest proportion, 12.2% were moderately depressed while the least proportion, 5.3% were severely depressed.

**Table 5:** Prevalence of anxiety and depression among the respondents

Variable	Frequency (n=450)	Percent (%)
**Prevalence of anxiety**		
Normal	286	63.6
Mild depression	67	14.9
Moderate depression	76	16.9
Severe depression	21	4.7
		
**Anxiety**		
Yes	164	36.4
No	286	63.6
		
**Prevalence of depression**		
Normal	312	69.3
Mild anxiety	59	13.1
Moderate anxiety	55	12.2
Severe anxiety	24	5.3
		
**Depression**		
Yes	138	30.7
No	312	69.3

Table 6 shows the correlation matrix of variables. There was a weak positive correlation between suicide and age in years, increases in suicidal ideation correlate with increases in age and this was found to be statistically significant, (n=450, r=0.107, p=0.023). There was also a weak positive correlation between suicidal ideation and depression, increases in depression correlates with increases in suicidal thoughts and this was found to be statistically significant, (n=450, r=0.260, p<0.001).

**Table 6:** Correlation matrix of variables

	Correlation co-efficient r, p value, (n=450)			
	Age in years	Depression	Anxiety	Suicide
Age in years	1			
				
Depression	r=0.157	1		
	p=0.001			
Anxiety	r=0.227	r=0.801	1	
	p<0.001	p<0.001		
Suicide	r=0.107	r=0.260	r=0.203	1
	p=0.023	p<0.001	p<0.001	

Table 7 shows the factors associated with suicide among the respondents. The prevalence of suicidal ideation among adolescents who were <15 years was 8.0% while those who were between 15 – 18 years was 13.5% and those more than 19 years of age was 22.5%. The prevalence of suicidal ideation among adolescents with depression is 21.7% and 78.3 % for hose without depression while the prevalence of suicidal ideation among adolescents with anxiety was 20.1% and 79.9 % for those without anxiety. The prevalence of suicidal ideation among the poorest half was 16.4% and 10.2% in the richer half. The respondents who were 19 years and above had the proportion of respondents who had suicidal intentions, 22.5% while those who were 15 years had the least, 8.0% and the difference in proportions was found to be statistically significant, (*χ*^2^=6.440, p=0.040). A significantly higher proportion of females, 16.6% had suicidal intentions when compared with the males, 7.1%, (*χ*^2^=7.958, p=0.005). A significantly higher proportion of respondents who had depression, 21.7% had suicidal intentions when compared with those who were not, (*χ*^2^=12.170, p<0.001). Similarly, a significantly higher proportion of respondents who had an anxiety disorder, (20.1%) had suicidal thoughts when compared with those who were not, 9.4%, (*χ*^2^=10.291, p=0.001).

**Table 7:** Factors affecting suicidal ideation among the respondents

Variable	Suicide severity rating		*χ* ^2^	p value
	scale Yes N (%)	(n=450) No N (%)		
**Age of respondents**				
<15 years	8 (8.0)	92 (92.0)	6.440	0.040
15 – 18 years	40 (13.5)	257 (86.5)		
≥19 years	12 (22.5)	41 (77.4)		
				
**Gender**				
Male	11 (7.1)	144 (92.9)	7.958	0.005
Female	49 (16.6)	246 (83.4)		
				
**Location**				
Urban	29 (12.1)	211 (87.9)	0.695	0.404
Rural	31 (14.8)	179 (85.2)		
				
**Educational attainment** **of Mother**				
Primary education and less	13 (12.6)	90 (87.4)	0.201	0.905
Secondary education	28 (14.1)	170 (85.9)		
Tertiary education	19 (12.8)	130 (87.2)		
				
**Family Socio-economic** **status**				
Low socio-economic class	37 (16.4)	188 (83.6)	3.769	0.052
High socio-economic class	23 (10.2)	202 (89.8)		
				
**Depression**				
Yes	30 (21.7)	108 (78.3)	12.170	<0.001
No	30 (9.6)	282 (90.4)		
				
**Anxiety disorder**				
Yes	33 (20.1)	131 (79.9)	10.291	0.001
No	27 (9.4)	259 (90.6)		

**Adjusted odds ratio, 95% confidence interval

NA Not applicable

Table 8 shows the predictors of Suicide among the respondents. The respondents who were less than 15 years were three times less likely to have the suicidal ideation when compared with those who were 19 years or more, (AOR=0.3, 95%CI: 0.1-0.9). Similarly, the respondents aged 15-18 years were 2.5 times less likely to have the suicidal ideation when compared with those who were 19 years and above, (AOR=0.4, 95%CI: 0.2-0.9). The respondents who were depressed were 1.9 times more likely to have a suicidal ideation when compared with those who were not depressed, (AOR=1.9, 95%CI: 0.8-1.9). The respondents who were anxious were 1.4 times more likely to have a suicidal ideation when compared with those who were not anxious, (AOR=1.4, 95%CI: 0.7-2.8).

**Table 8:** Predictors of suicide among the respondents using Hierarchical logistic regression analysis

Variable	Model 1 Adjusted OR (95% C. I.)	P- value	Model 2 Adjusted OR (95% C. I.)	P-value
**Age of respondents**				
<15 years	0.3 (0.1-0.8	0.020	0.3 (0.1-0.9)	0.034
15 – 18 years	0.5 (0.2-1.1)	0.059	0.4 (0.2- 0.9)	0.028
≥19 years	1		1	
				
**Gender**				
Male	0.4 (0.2- 0.8)	0.008	0.5 (0.2-1.1)	0.474
Female	1		1	
				
**Location**				
Urban	1.4 (0.7-2.7)	0.370	1.6 (0.8- 3.2)	0.215
Rural	1		1	
				
**Educational attainment of** **Mother**				
Primary education and less	1.1 (0.5-2.2)	0.926	0.9 (0.4- 2.1	0.889
Secondary education	1.2 (0.7-2.4)	0.514	1.2 (0.6-2.2)	0.667
Tertiary education	1		1	
				
**Family Socio-economic status**				
Low socio-economic class	1.8 (0.9-3.6)	0.069	1.8 (0.9-3.6)	0.071
High socio-economic class	1		1	
				
**Depression**				
Yes	-	-	1.9 (0.9- 3.8)	0.280
No	-	**-**	1	
				
**Anxiety disorder**				
Yes	-	-	1.5 (0.7-3.0)	0.066
No	-	**-**	1	
				
**Model summary**	**Model 1**		**Model 2**	
-2 Log-likelihood	335.205		336.457	
Nagelkerke R square	0.073		0.107	
Hosmer and Lemeshow test	0.601		0.167	
Omnibus test of model coefficients	18.202, (p= 0.011)		8.748, (p=0.013)	

Adjusted odds ratio, 95% confidence interval

## Discussion

This study aimed to determine the pattern of adolescent depression/anxiety and their association with suicidal ideation. It also determines the predictors of suicidal ideation among adolescents.

The study showed the prevalence of depression among secondary school adolescents as 30.7% (with the least proportion of 5.3% being severely depressed), and anxiety as 36.4% (with the least proportion, 4.7% having severe anxiety). There had been rising trends of adolescent depression and anxiety over the decades. 22-24 For instance Michael et al 23 noted the prevalence of past-year major(severe) depressive episode to have increased by 7.7% from 2009 to 2019. These rising trends could be explained by the difficult economic situations coupled with political instabilities that are currently witnessed in the country.

This study had shown documented evidence of adolescent suicidal ideation among secondary school adolescents. Although a minor proportion (8.4%) of the adolescents in this study have wished themselves dead or even sleep and did not wake up while a few (8.7%) had worked out details of how to kill themselves. However, these figures seen above are higher than that seen in Nigerian study where a prevalence of suicidal ideation was noted as 6.1% 24 and attempt at suicidal as 2.8%. 24 The prevalence of suicidal ideation varies within each country. For instance, Ghana has a rate of 0.1 per 100,000 while Namibia had 100 per 100,000.^15^ Lifetime prevalence estimates for suicide attempts vary from 0.7% in Nigeria.^15^ to 6.0% in Liberia. 15 As seen in this study suicidal ideation has no gender prediction. However, studies have also reported higher rates of suicide in males with most reporting a male to female ratio of at least 3:1.^25^

A significantly higher proportion of females had suicidal intentions when compared with the males as seen in this reportage. It is important to note that whereas females have a higher tendency to suicidal thoughts, males seem to have higher completed suicide rates than females.^25^ This is an essential social facts in suicidology, however, the underlying reasons remain a subject of debate.^25^ The Breed's 5 “components of a basic suicide syndrome” explains the gender-related differences in suicide attempts.^26^ It stated that independence and decisiveness are valued by males while females are more interdependent, and they consult their peers and readily accept help.^25^ The female gender put a lot into perspective before taking a major decision and they can easily change their mind. 25 Furthermore, the socialization theory suggests that both genders tend to adopt self-destructive behaviours depending on their socio-cultural backgrounds; therefore, suicide attempts and ideation would be “more acceptable” among females but suicide itself will be seen among males.^25^

There is an increased rate of anxiety, depression, and suicidal ideation with increasing age as seen in the current study. It is also revealed in the present study that adolescents who were less than 15 years were three times less likely to have the suicidal ideation when compared with those who were 19 years or more, similarly adolescents who were aged 15-18 years were 2.5 times less likely to have the suicidal ideation when compared with those who were 19 years and above. In Africa, the lowest suicide rates were reported among adolescents who were below the age of 25 and a low prevalence of suicide has been reported in children under 15 years (≤0.5 per 100,000). 15 The increasing suicide rates with age could be explained by the higher suicide rates of adolescents in the mid-life period, it could also be due to social or historical events occurring during the period widely known as “the period effect”. 15

This present study showed that the prevalence of suicidal ideation among the poorest half was higher than that of the richer half. Studies have shown that adolescents with low socioeconomic class had a higher tendency to commit suicide compared to those with a higher class. Besides, socioeconomic disparities in suicide have been an area of debate. For example, a study in Asia, 26-28 Li et al 29, North America 30 Europe 31-36, Australia and New Zealand^34^ and a cross-comparative study of fourteen European countries^37^ showed that subjects from lower socioeconomic groups were found to be more likely to die by suicide than those in higher socioeconomic groups.^37^ Furthermore, suicide rates were noted to be highest among less educated people. 38-40

The present study had shown that adolescents with depression were 1.9 times more likely to have a suicidal ideation when compared with those who don't have anxiety. Similarly, adolescents with anxiety were 1.4 times more likely to have a suicidal ideation when compared with those who were not depressed. Sub-threshold depression and sub-threshold anxiety are associated with an increased burden of suicide risk. Balazs et al 40 have noted that if incorporated in the diagnostic modules, the disorders of anxiety and depression could provide a bridge between categorical and dimensional diagnostic models. The association between depression and suicide attempts or ideation cannot be overemphasized, however, the association of suicidal activity with anxiety disorders is not well elaborated. Besides, studies have shown that some pattern of major depression contains heterogeneous subtypes that do not share the same suicidal ideation. 38 Furthermore, the import of anxiety disorders in increasing suicidal risk still remains a matter of controversy. 38Although it is documented that the association between depression and anxiety disorders has a complementary role in increasing the suicidal ideation. 38 We noted no significant difference in suicidal ideation among adolescents who were rural or urban dwellers. Adolescent suicide rates are noted to exhibit wide geographic differences with the highest rates of suicide fatality in the rural areas.^38^ The causes of this rural dominance in a study were not ascertained, however the possibility that urban adolescents are more likely than rural counterparts to access psychological/psychiatric care may explain this disparity.

## Strength of this study

It will be very useful as reference point for studies on adolescent suicide in this vicinity, especially for the fact that it is enriched with a very large sample size of adolescents drawn from several secondary schools in Nigeria.

## Conclusion

There was a positive correlation between suicide and age in years. Importantly, a significantly higher proportion of respondents who had depression, 21.7% had suicidal ideation when compared with those who were not, among those with an anxiety disorder, 20.1% had suicidal ideation when compared with those who were not. Moreover, the respondents who were 19 years and above had suicidal ideation, and the difference in proportions was found to be statistically significant. Finally, a significantly higher proportion of females, 16.6% had suicidal intentions when compared with the males, 7.1%. There is a higher prevalence of risk of suicide among urban than rural dwellers. The prevalence suicidal ideation among the poorest half was higher than that of the richer half.

## Clinical implications

These findings suggest that there are variable prevalence of depression, anxiety and suicidal ideation among adolescents. This prevalence also varies between social class and between urban and rural dwellers. The major clinical implication from the study is that gathering information about the prevalence of depression and anxiety of the adolescent can inform the assessment of suicide ideation. This could further help to appropriate referral of those adolescents with suicidal ideation for proper psychiatric evaluation so as to avert complete actual suicide. The findings obtained from this work will also guide clinical decision making regarding the level of mental health interventions the adolescent may require. In addition, adolescents who presented with a history of suicidal behavior require some psychiatric intervention depending on the level and severity of their intent, underlying risk factors, and their resilience and social support system.

## Limitation

This study is limited by the fact the work was done mainly in the southeastern part of the country which may not reflect the true picture of anxiety, depression and suicidal ideation the entire country. Some adolescents did not feel comfortable sharing sensitive information with the research assistants. Co-founders such as current struggles with academics/poor academic performance, chronic ill health such as sickle cell disease, childhood rheumatoid arthritis was not considered in the course of this work.

## Recommendation

A high index of suspicion of at-risk-group especially depression and anxiety.

## Data Availability

Data are however available from the authors upon reasonable request and with permission of the corresponding Author.
